# Green Synthesis and Catalytic Activity of Silver Nanoparticles Based on *Piper chaba* Stem Extracts

**DOI:** 10.3390/nano10091777

**Published:** 2020-09-08

**Authors:** Md. Mahiuddin, Prianka Saha, Bungo Ochiai

**Affiliations:** 1 Department of Chemistry and Chemical Engineering, Graduate School of Science and Engineering, Yamagata University, Jonan 4-3-16, Yonezawa, Yamagata 992-8510, Japan; 2 Chemistry Discipline, Faculty of Science, Engineering and Technology, Khulna University, Khulna 9208, Bangladesh; prianka24saha@gmail.com

**Keywords:** silver nanoparticles, *Piper chaba*, green synthesis, catalytic activity

## Abstract

A green synthesis of silver nanoparticles (AgNPs) was conducted using the stem extract of *Piper chaba*, which is a plant abundantly growing in South and Southeast Asia. The synthesis was carried out at different reaction conditions, i.e., reaction temperature, concentrations of the extract and silver nitrate, reaction time, and pH. The synthesized AgNPs were characterized by visual observation, ultraviolet–visible (UV-vis) spectroscopy, dynamic light scattering (DLS), scanning electron microscopy (SEM), transmission electron microscopy (TEM), x-ray diffraction (XRD), energy dispersive x-ray (EDX), and Fourier transform infrared (FTIR) spectroscopy. The characterization results revealed that AgNPs were uniformly dispersed and exhibited a moderate size distribution. They were mostly spherical crystals with face-centered cubic structures and an average size of 19 nm. The FTIR spectroscopy and DLS analysis indicated that the phytochemicals capping the surface of AgNPs stabilize the dispersion through anionic repulsion. The synthesized AgNPs effectively catalyzed the reduction of 4-nitrophenol (4-NP) and degradation of methylene blue (MB) in the presence of sodium borohydride.

## 1. Introduction

In the last decade, green synthesis of metal nanoparticles has been exponentially increasing because of its various applications, such as electronics, catalysis, chemistry, energy, and medicine [1,2,3]. In particular, silver nanoparticles (AgNPs) have many important applications in several fields, such as high sensitivity biomolecular detection and diagnostics [4,5,6], antimicrobials [7,8,9,10,11,12,13], catalysis [14,15,16,17,18], optics [19], biomedicine [20], medicine [21,22], as well as food and cosmetic industries [23,24].

Conventional methods for manufacturing AgNPs, such as chemical reduction, electrochemical process, photochemical reduction, laser ablation, hydrothermal method, microemulsion method, radiation-induced, radiolytic reduction, sonochemical reduction, pyrolysis, and lithography [13,25,26,27] are expensive, environmentally toxic, and/or hazardous. For example, the most typical chemical synthesis of AgNPs requires reducing agents such as sodium borohydride, hydroxylamine, sodium citrate, and hydrazine with environmentally undesirable solvents in some cases [28]. In most cases, this produces byproducts that are harmful to the environment [2]. Thus, green synthesis methods for AgNPs are highly required. The green methods should reduce the need for high pressure, high temperature, and toxic chemicals. They should also be simple (including only one step), rapid, cost-effective, and reproducible [1]. Extracts from various plants, such as *Solanum nigrum* [8], *Coleus aromaticus* [9], *Alpinia katsumadai* [10], *Acalypha indica* [11], *Clitoria ternatea*, *Cinnamon zeylanicum* [12], *Breynia rhamnoides* [18], *Azadirachta indica* [29], *Lantana camara* [30], olive (*Olea europaea*) leaf [31], *Thymbra spicata* [32], *Gmelina arborea* [33], *Skimmia laureola* [34], *Arbutus unedo* [35], *Viburnum opulus L.* [36], *Pice abies L.* [37], green coffee (*Coffea arabica, Coffea canephora, and Coffea liberica*) bean [13], and clove (*Syzygium aromaticum*) [38] are promising substances for a green and facile synthesis of AgNPs.

**Figure 1:**
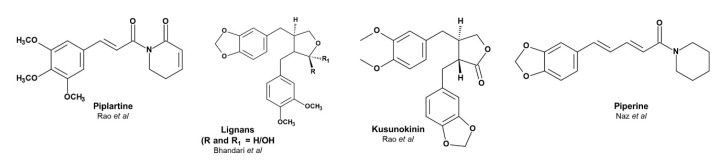
Examples of compounds in the stem extract of *Piper chaba*.

*Piper chaba* is a common plant known as chui jhal or choi jhal and belongs to the Piperaceae family. It is a very cheap and edible plant found in abundance in South and Southeast Asia [39]. Beyond edible purposes, it is used in various applications, such as in analgesic, antibacterial, antioxidant, and hypotensive functions [39,40,41]. *Piper chaba* contains various compounds, among which alkaloids and lignan are predominant [42,43,44]. Figure 1 shows examples of these compounds. The polyphenolic, conjugated, and hemiacetal structures of these compounds have the potential to reduce Ag^+^ to Ag^0^. The availability, cost-effectiveness compared to other plant sources, edibility, and the presence of various functional compounds made us enthusiastic to utilize *Piper chaba* as a source of AgNP synthesis.

This study aimed to synthesize AgNPs using the stem extract of *Piper chaba* and to investigate the effect of temperature, reaction time, concentration of AgNO_3_, quantity of plant extract, and pH on the synthesis process. The synthesized AgNPs were characterized by ultraviolet–visible (UV-vis) spectroscopy, dynamic light scattering (DLS), zeta potential measurements, scanning electron microscopy (SEM), transmission electron microscopy (TEM), x-ray diffraction (XRD), energy dispersive x-ray spectroscopy (EDX), and Fourier transform infrared (FTIR) spectroscopy. The catalytic activity of the synthesized AgNPs was examined during the reduction reaction of 4-nitrophenol (4-NP) and during the degradation of methylene blue (MB) in the presence of NaBH_4_.

## 2. Materials and Methods

### 2.1. Collection of Plant Materials

The stem of *Piper chaba* was freshly collected from Khulna District, Bangladesh. The stem was thoroughly washed with deionized distilled water, cut into small pieces, and air-dried at room temperature.

### 2.2. Preparation of Plant Extract

Finely cut stems (approximately 20 g) were placed in a round-bottom flask containing deionized distilled water (200 mL) and boiled for 40 min. The extract was cooled down, filtered using a filter paper, and stored at 4 °C for further use.

### 2.3. Materials

AgNO_3_ and 4-NP were purchased from Merck Chemicals (Merck KGaA, Darmstadt, Germany) and Tokyo Chemical Industry Co. Ltd. (Tokyo, Japan), respectively, whereas MB and sodium borohydride were obtained from Kanto Chemical Co. Inc. (Tokyo, Japan).

### 2.4. Measurements

UV-vis spectroscopic analysis was carried out on a JASCO (Tokyo, Japan) V-730 series and a UVD-3200 (LABOMED, CA, USA) spectrometer. Absorption spectra were recorded at a resolution of 1 nm within 200–800 nm. The hydrodynamic size and zeta potential were measured through DLS analysis conducted on a Malvern (Malvern, UK) Zetasizer Nano ZS instrument. FTIR spectra were recorded on a JASCO (Tokyo, Japan) FT/IR-460 plus spectrometer using a KBr pellet with a scan rate of approximately 4 cm^−1^ s^−1^ at 25 °C. SEM measurements and EDX analysis were conducted on a Hitachi (Tokyo, Japan) SU-8000 microscope at accelerating voltages of 10 and 15 kV. TEM measurements were conducted on a TEM-2100F (JEOL, Tokyo, Japan) field emission electron microscope. XRD analysis was conducted on a Rigaku (Tokyo, Japan) Ultima IV RINT D/max-kA spectrometer with Cu-Kα radiation.

### 2.5. Green Synthesis of AgNPs

An aqueous solution of AgNO_3_ (1 mM, 100 mL) was prepared in a volumetric flask, and the flask was covered with carbon paper to prevent the autoxidation of silver. The aqueous stem extract of *Piper chaba* (4 mL) was placed in a conical flask. Then, the freshly prepared AgNO_3_ aq. (1 mM, 80 mL) was added to the conical flask. The mixture was heated in an oil bath at 60 °C for 30 min under constant stirring. After this, the color of the solution changed from colorless to reddish brown. The resulting suspension was stored at room temperature for 48 h. The reaction mixture containing AgNPs was then centrifuged at 13,000 rpm for 30 min, and the precipitate was thoroughly washed three times with sterile distilled water to remove impurities.

### 2.6. Catalytic Reduction of 4-Nitrophenol to 4-Aminophenol

The photo-catalytic reduction of 4-NP was performed in a quartz cuvette (height = 4 cm and optical path length = 1 cm). Aqueous solutions of NaBH_4_ and 4-NP at concentrations of 600 ppm and 10 ppm, respectively, were used in the process and they were stored at 4 °C before use. The photo-catalytic reduction was carried out by mixing the 4-NP solution (1 mL), NaBH_4_ solution (2 mL), and colloidal suspension of AgNPs (53.9 mg/L, 100 μL) in the cuvette. The time course of 4-NP consumption was monitored by optical absorbance at 401 nm. The control experiment without AgNPs was also carried out through an identical procedure.

### 2.7. Degradation of MB

The degradation of MB was performed similarly to the reduction of 4-NP. The degradation reaction was studied by mixing an aqueous solution of MB (2 ppm, 2 mL), NaBH_4_ solution (600 ppm, 1.5 mL), and colloidal suspension of AgNPs (53.9 mg/L, 100-μL) in the cuvette. The time course of MB consumption was monitored by optical absorbance at 663 nm. The control experiment without AgNPs was also carried out through an identical procedure.

## 3. Results and Discussion

### 3.1. Formation of AgNPs

**Figure 2:**
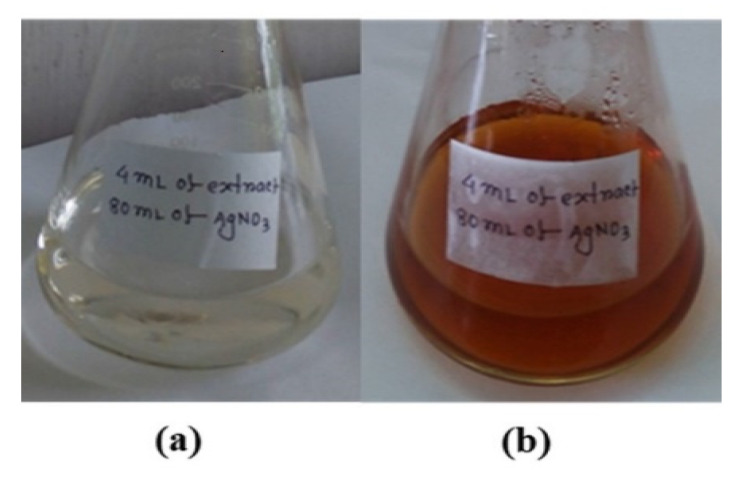
Images of (**a**) the mixture of the *Piper chaba* stem extract and solution of AgNO_3_ (1 mM) and (**b**) the mixture after a 30-min reaction.

**Figure 3:**
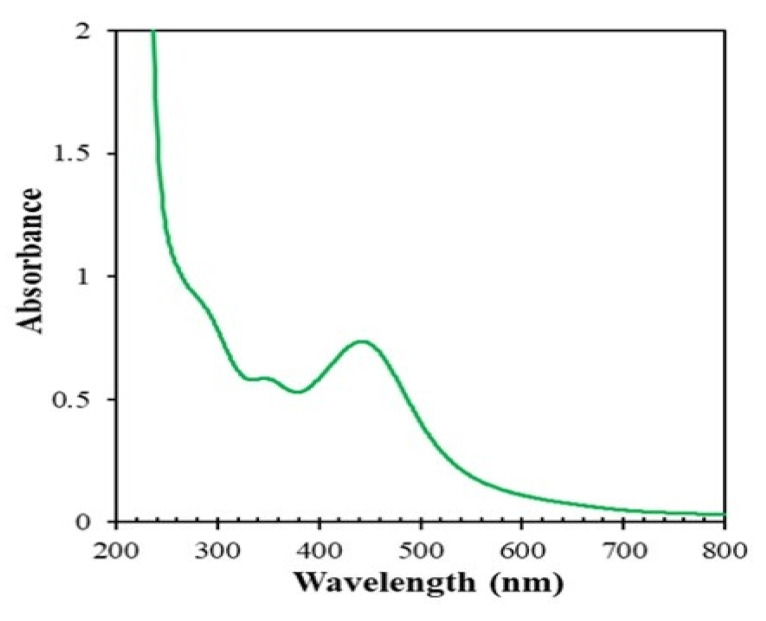
Ultraviolet–visible (UV-vis) absorption spectrum of reaction mixtures of AgNO_3_ (1 mM, 40 mL) and *Piper chaba* extract (2 mL, 100 g/L) at 60 °C and pH = 7 after 30 min.

AgNPs were synthesized by in situ reduction followed by a coating with the stem extract of *Piper chaba*. The initial mixture was colorless (Figure 2a) and its color changed to reddish brown after the reaction (Figure 2b). The formation of AgNPs in the medium was confirmed by a surface plasmon resonance (SPR) band and a UV-vis absorption peak corresponding to AgNPs at approximately 445 nm (Figure 3). The resulting AgNPs dispersed stably over 12 weeks, while AgNPs prepared by conventional reduction with NaBH_4_ in the absence of stabilizing agent were precipitated within 12 h.

### 3.2. Effect of the Synthesis Conditions

The key factors that affect the synthesis of AgNPs, i.e., temperature, concentration of AgNO_3_, concentration of the plant extract, reaction time, and pH, were examined.

#### 3.2.1. Effect of Temperature

**Figure 4:**
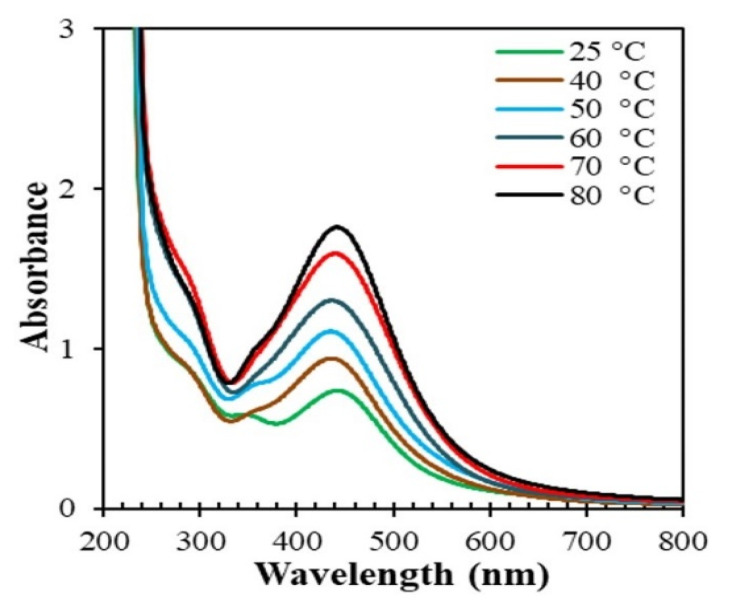
Absorption spectra of reaction mixtures of AgNO_3_ (1 mM, 40 mL) and *Piper chaba* extract (2 mL, 100 g/L) at pH = 7 after 1 h at different temperatures.

Figure 4 shows the absorption spectra of the reaction mixtures consisting of the stem extract of *Piper chaba* and AgNO_3_ after 1 h of reaction at different temperatures. The absorption of SPR increased with temperature. The change in the color of the solutions was accelerated at higher temperatures, indicating the rapid reduction of Ag^+^ to form AgNPs. Beyond 60 °C, the absorption maxima slightly shifted to longer wavelengths, which probably originated from the aggregation of AgNPs in uncontrolled reactions. This result suggests that 60 °C is the optimum temperature to obtain high-quality AgNPs.

#### 3.2.2. Effect of AgNO_3_ Concentration

**Figure 5:**
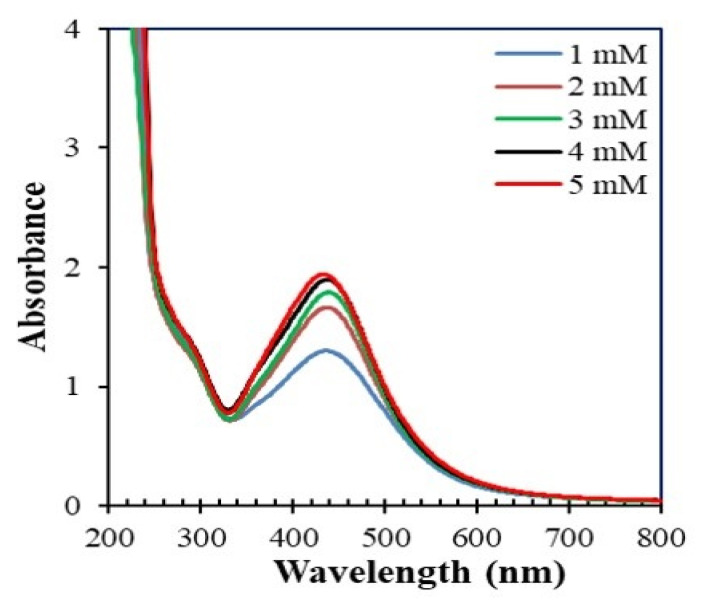
Absorption spectra of the reaction mixtures consisting of *Piper chaba* extract (100 g/L, 2 mL) and AgNO_3_ (1 to 5 mM, 40 mL) at 60 °C and pH = 7 after 1 h of reaction.

Figure 5 shows the absorption spectra of the reaction mixtures consisting of the stem extract of *Piper chaba* and AgNO_3_ after 1 h of reaction at 60 °C using different concentrations of AgNO_3_ solution. The absorbance increased, and a blue shift occurred with the increase in the AgNO_3_ concentration. Beyond 2.0 mM, the intensity stabilized due to the insufficient amount of the plant extract needed to react with Ag^+^. Thus, to obtain optimum quality and quantity of AgNPs, lower concentrations of AgNO_3_ should be used.

#### 3.2.3. Effect of Plant Extract Concentration

**Figure 6:**
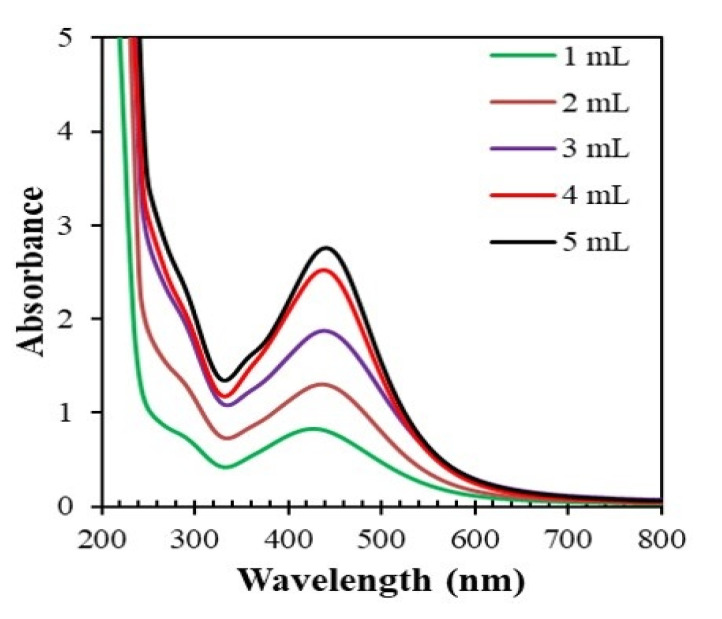
Absorption spectra of reaction mixtures of *Piper chaba* extract (100 g/L, 1–5 mL) and AgNO_3_ (1 mM, 40 mL) after 1 h of reaction at 60 °C and pH = 7.

Figure 6 shows the absorption spectra of the reaction mixtures consisting of various concentrations of the *Piper chaba* stem extract and 1 mM aqueous solution of AgNO_3_ (40 mL) after 1 h of reaction at 60 °C and neutral conditions. The peak intensity increased, and a red shift occurred with the increase in the plant extract concentration. The increased intensity indicates an enhanced production and faster growth of AgNPs. However, the red shift originated from an uncontrolled reduction by the plant extract excess. Thus, lower concentrations of the *Piper chaba* stem extract are suitable to obtain a quality product.

#### 3.2.4. Effect of Reaction Time

**Figure 7:**
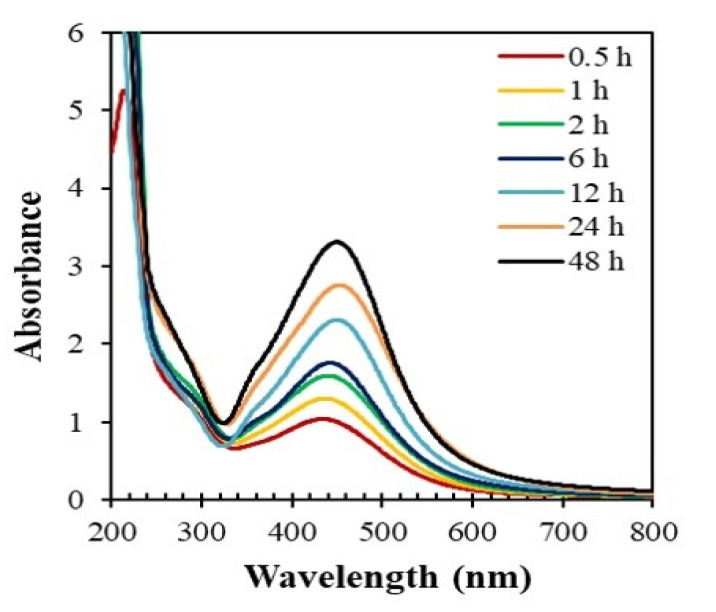
Time-dependent absorption spectra of the reaction mixtures consisting of AgNO_3_ (1 mM, 40 mL) and *Piper chaba* extract (100 g/L, 2 mL) at 60 °C and pH = 7.

Figure 7 shows the time course of the absorption spectra of the reaction mixtures consisting of the *Piper chaba* stem extract (2 mL) and AgNO_3_ solution (40 mL, 1 mM) at 60 °C and pH = 7. The intensity of the absorption increased with the reaction time, indicating the gradual formation of AgNPs. On the other hand, the absorption maxima gradually shifted toward longer wavelengths after 6 h, which indicates the agglomeration of AgNPs to form larger particles. Thus, a reaction time frame from 1 to 6 h was found suitable to produce good yields of quality AgNPs.

#### 3.2.5. Effect of pH on the Formation of AgNPs

**Figure 8:**
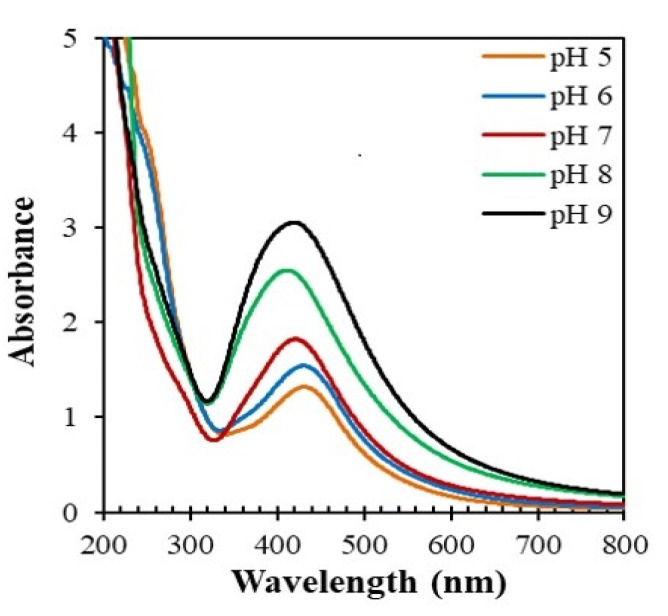
pH-dependent absorption spectra of the reaction mixtures of AgNO_3_ (1 mM, 40 mL) and *Piper chaba* extract (100 g/L, 2 mL) after the reaction for 1 h at 60 °C.

The pH of the reaction medium played a pivotal role during the formation of AgNPs. The pH change affects the shape and size of the nanoparticles because the pH alters the electrostatic states of the substances in the plant extract, which affects the stabilizing abilities and subsequently the growth of the nanoparticles [31]. Figure 8 illustrates the absorption spectra of the reaction mixtures consisting of the stem extract of *Piper chaba* (2 mL) and AgNO_3_ (40 mL, 1 mM) at different pH. The absorption of SPR increased and a blue shift occurred with the increase in pH. This suggests that lower pH predominantly induced the aggregation of AgNPs rather than the nucleation to form new particles. At a higher pH, many anionic functional groups can bind with the silver ions to form a large number of nuclei for AgNPs which results in AgNPs with a smaller diameter. Sathishkumar et al. [12] reported a similar pH effect as they found that silver species were rapidly reduced at elevated pH. Thus, neutral or basic conditions are optimum for the reduction reaction. The pH of the plant extract and AgNO_3_ mixture was seven, therewith neutral conditions are advantageous for facilitating the reaction. Thus, the AgNPs used for further analysis were synthesized using plant extract (2 mL) and AgNO_3_ (1 mM, 40 mL) at 60 °C and pH = 7 for 1 h.

### 3.3. Structure of AgNPs

**Figure 9:**
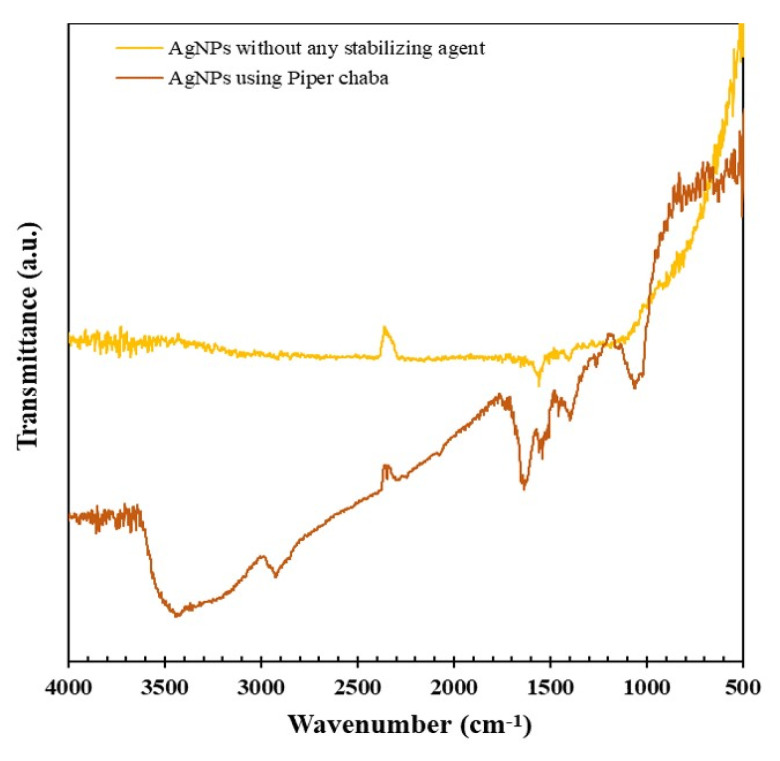
Fourier transform infrared (FTIR) spectra of silver nanoparticles (AgNPs) synthesized using AgNO_3_ (1 mM, 40 mL) and *Piper chaba* extract (100 g/L, 2 mL) after the reaction for 1 h at 60 °C and pH = 7, and AgNPs synthesized without capping agent reduced by NaBH_4._

The structure of the organic components on the surface of AgNPs was investigated using FTIR spectroscopy (Figure 9). The spectrum AgNPs obtained using *Piper chaba* stem extracts shows a broad band at 3447 cm^−1^ assigned to the O–H and N–H stretching vibrations. The broad shoulder at the lower wavelength region implies that the O–H and N–H bonds form hydrogen bonds with the capping organic molecules and the surface of AgNPs. The band observed at 2925 cm^−1^ was attributed to the aliphatic C–H stretching. Signals were almost unobservable around 3000 cm^−1^ and 1700–1750 cm^−1^. These can be ascribed to negligible contents of aromatic and alkenyl protons as well as ester and ketone moieties. The broad band at 1636 cm^−1^ corresponded to the stretching vibration of C=O in amides, which were found in *Piper chaba* [45,46,47]. The bands arising from C–O–H bending and C–O stretching were observed at 1397 cm^−1^ and 1064 cm^−1^, respectively, which agree with polysaccharides commonly found in plants. This FTIR spectroscopic data confirm the plausible structures for capping substances. These characteristic FTIR peaks were not observable in the spectrum of AgNPs synthesized without any stabilizing agent [48], indicating that the capping substances originate from *Piper chaba* stem extracts.

**Figure 10:**
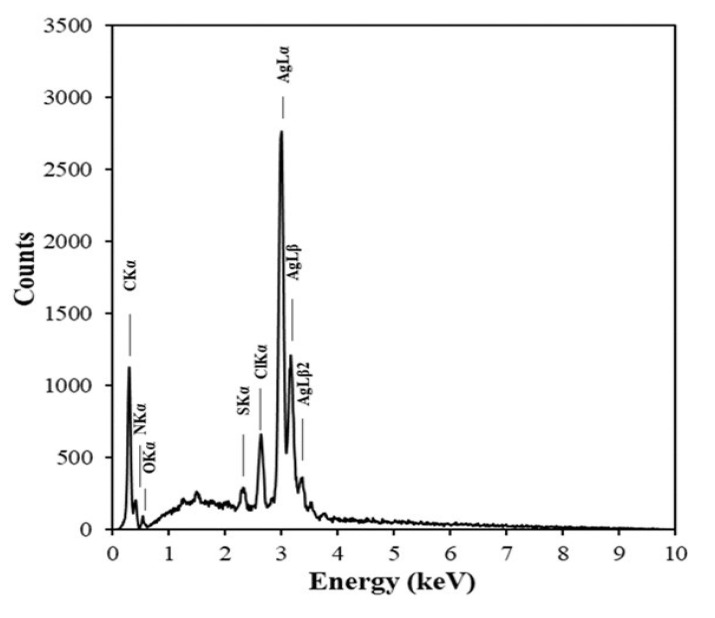
Energy dispersive x-ray (EDX) spectrum of AgNPs synthesized using AgNO_3_ (1 mM, 40 mL) and *Piper chaba* extract (100 g/L, 2 mL) at 60 °C and pH = 7 for 1 h.

Figure 10 illustrates the EDX spectrum of the green-synthesized AgNPs. The spectrum shows strong signals of Ag. The signals observed for C, N, O, S, and Cl can be attributed to the organic substances found in the plant extract. The weaker intensities of these signals imply the presence of a thin coating layer on AgNPs.

### 3.4. SEM and TEM Results

**Figure 11:**
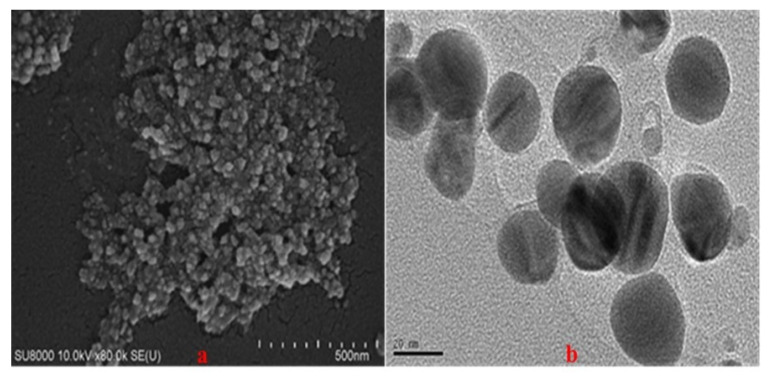
(**a**) Scanning electron microscopy (SEM) and (**b**) transmission electron microscopy (TEM) images of the AgNPs synthesized from AgNO_3_ (1 mM, 40 mL) and *Piper chaba* extract (100 g/L, 2 mL) at 60 °C and pH = 7 for 1 h.

The morphology and size of AgNPs were evaluated using SEM and TEM. Figure 11a shows the SEM image of the AgNPs. The AgNPs were found to be almost spherical with homogeneous morphology. The average diameter of the primary particles was 26 nm with a standard deviation and a coefficient of variation of 4.75%.

Figure 11b shows the TEM image of the AgNPs. The particles were found to be spherical with an average diameter of 19 nm. Isolated primary particles were also observed. These results are in good agreement with the SEM results.

### 3.5. XRD Analysis

**Figure 12:**
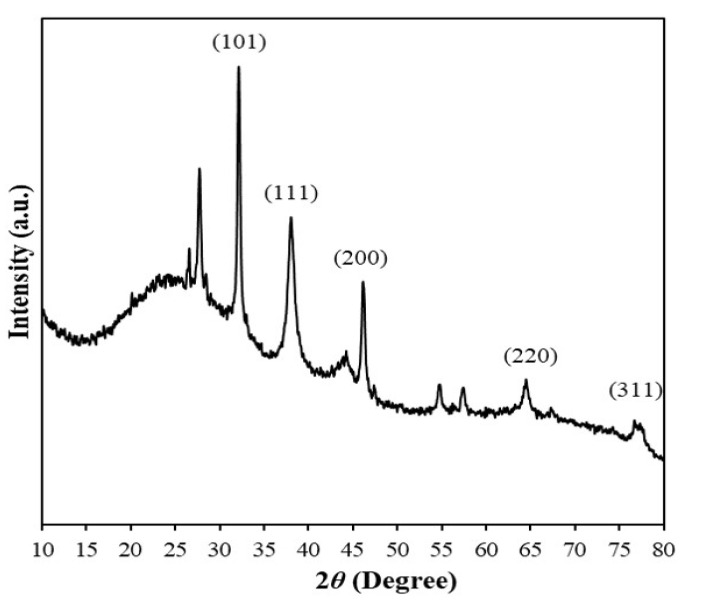
X-ray diffraction (XRD) pattern of AgNPs synthesized using AgNO_3_ (1 mM, 40 mL) and *Piper chaba* extract (100 g/L, 2 mL) at 60 °C and pH = 7 for 1 h.

Figure 12 shows the XRD pattern of dried AgNPs. The five distinct diffraction peaks with 2θ = 32.12°, 38.02°, 46.1°, 64.16°, and 75.36°, which can be assigned to the following lattice planes: (101), (111), (200), (220), and (311), respectively, in Ag(0) with the face-centered cubic structure (JCPDS file no. 84-0713 and 04-0783). The average size of nanocrystallites was estimated by the Scherrer formula as follows:

D=kλ/βcosθ

where *D* is the diameter of coherent diffraction domain, *k* is the shape constant (*k* = 1 for spherical domains), *λ* is the wavelength of the x-ray source (0.1541 nm), *β* is the full width at half maximum, and θ is the diffraction angle corresponding to the lattice plane (111). The average crystallite size calculated from the Scherrer equation is 20.9 nm, which is similar to the average size of AgNPs observed in the microscopic images. This suggests that the primary particles of AgNPs almost consisted of single crystallites.

A few unassigned peaks (26.56°, 27.7°, 54.68°, and 56.42°) were also observed, which could be attributed to the biomass residue capping AgNPs. This suggests that the crystallization of the bio-organic phase occurs on the surface of the silver nanoparticles [9,22].

### 3.6. DLS Study and Zeta Potential Study

**Figure 13:**
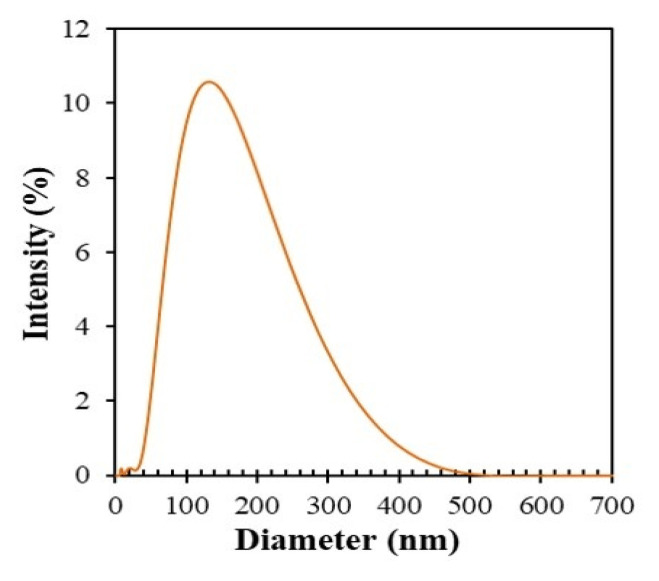
Dynamic light scattering (DLS) curve of AgNPs synthesized using AgNO_3_ (1 mM, 40.0 mL) and *Piper chaba* extract (2 mL, 100 g/L) at 60 °C at pH = 7 for 1 h.

DLS measurement was conducted to evaluate the hydrodynamic diameter of AgNPs (Figure 13). The average hydrodynamic diameter (*D*_h_) of AgNPs was found to be 122 nm, which is larger than the sizes obtained from SEM and TEM measurements. A similar difference was reported for AgNPs prepared from *Ekebergia capensis* and *Abelmoschus esculentus* L. [49,50]. DLS measurements evaluate NPs with a hydrated layer consisting of swollen organic moieties on the surface of AgNPs in Brownian motion [51,52]. The hydrated layer and the Brownian motion resulted in the larger *D*_h_. The polydispersity index was found to be 0.221. This moderate value probably originated from various components in the plant extract that was covering the nanosized AgNPs.

The zeta potential was −22.2 mV. This sufficiently negative zeta potential confirms the good dispersibility of AgNPs caused by electrostatic repulsion.

### 3.7. Application of the Synthesized AgNPs in the Reduction of 4-Nitrophenol (4-NP)

**Figure 14:**
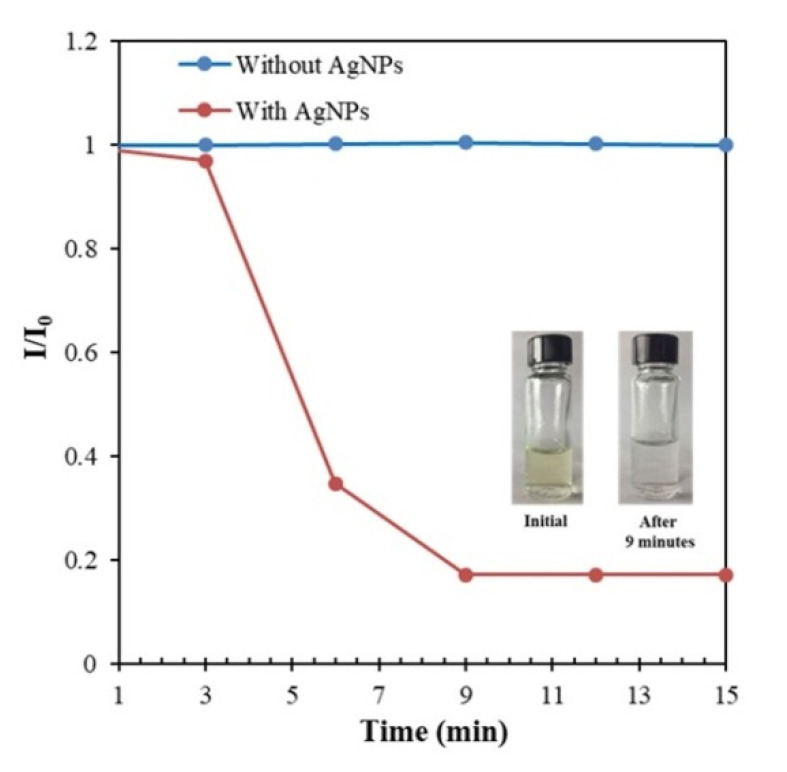
Optical images and time-dependence of the absorbance at 401 nm for the catalytic reduction of 4-NP by NaBH_4_ in the presence of AgNPs. Conditions: 4-NP = 10 ppm; catalyst = 53.9 mg/L; NaBH_4_ = 600 ppm; temperature = 25 °C.

The ability of the synthesized AgNPs to act as catalysts was examined. The reduction of 4-NP to 4-aminophenol by NaBH_4_ was selected as a model for the catalytic reduction. The catalytic process was monitored using UV-vis spectroscopy. The original absorption peak of 4-NP was observed at 316 nm. Upon addition of freshly prepared aqueous NaBH_4_ solution, the absorption maxima instantaneously shifted from 316 to 401 nm in the presence of AgNPs, and the color of the solution changed from light yellow to yellowish green, indicating the formation of the 4-nitrophenolate ion. When no AgNPs were used, the color and optical absorption did not change until after 1 h of reaction. The reduction of 4-NP in the presence of NaBH_4_ is a thermodynamically feasible process, but it is kinetically restricted in the absence of catalysts [14]. The kinetics of the reduction reaction was monitored using UV-vis spectroscopy. In the presence of AgNPs, 4-NP was almost completely reduced in approximately 9 min (Figure 14), which is comparable to reported fine AgNPs with identical sizes [13,38]. As the reduction reaction of 4-NP progressed, the intensity of the peak at 401 nm decreased, and a new peak corresponding to the 4-AP formation simultaneously occurred at 296 nm [13,16,17,18].

### 3.8. Application of the Synthesized AgNPs in the Catalytic Degradation of MB

**Figure 15:**
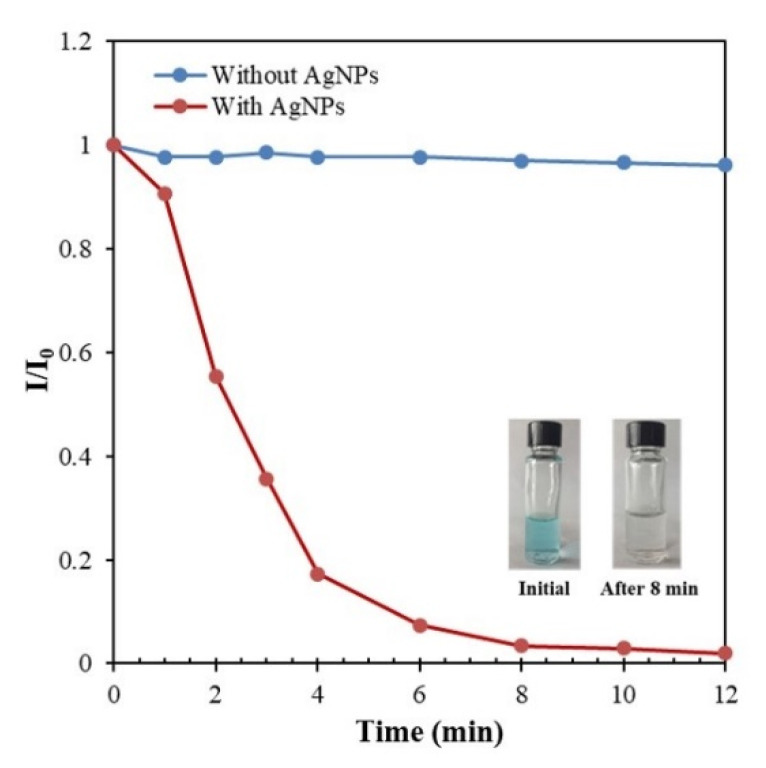
Optical images and time-dependence of the absorbance at 663 nm for the catalytic degradation of methylene blue (MB) dye by NaBH_4_ in the presence of AgNPs. Conditions: MB = 2 ppm; catalyst = 53.9 mg/L; NaBH_4_ = 600 ppm; temperature = 25 °C.

**Figure 16:**
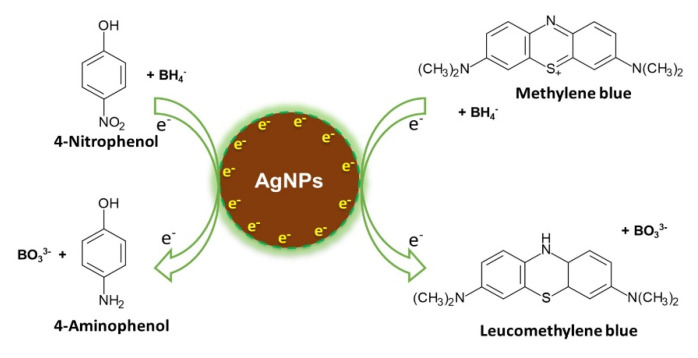
Schematic illustration of the catalytic process occurring on AgNPs.

The catalytic degradation of dyes in the presence of NaBH_4_ was examined as another model reaction to confirm the catalytic activity of AgNPs using MB. The catalytic process was monitored by UV-vis spectroscopy. Similar to 4-NP, NaBH_4_ needs a catalyst to react with MB. Thus, the color of the solution did not change for more than 1 h without AgNPs. With the addition of as-synthesized AgNPs to the reaction mixture, the catalytic reduction of dyes immediately occurred. During the degradation of MB, the color of the solution changed from blue to colorless after 8 min, which is also comparable to previously reported fine AgNPs [17,33]. The original absorption peak at 663 nm progressively decreased with time, which confirms the catalytic activity of the synthesized AgNPs (Figure 15). Figure 16 schematically illustrates the mechanism of the catalytic processes for degradation of both 4-NP and MB using NaBH_4_ as the reductant and AgNPs as the catalyst [16].

## 4. Conclusions

In this study, a successful, rapid, and green synthesis of AgNPs was achieved using *Piper chaba* stem extract as an effective reducing and capping agent. The parameters affecting the synthesis of AgNPs (i.e., temperature, concentration of AgNO_3_, quantity of plant extract, reaction time, and pH) were studied. The formation of AgNPs was confirmed by the SPR peak at 445 nm and XRD pattern. The obtained AgNPs were spherical and monocrystalline, ranging in size from 20 nm to 26 nm. The capping of the obtained AgNPs was confirmed using FTIR spectroscopy. The DLS measurement showed that AgNPs were uniformly dispersed and exhibited a moderate size distribution. The catalytic activity of the green-synthesized AgNPs was confirmed when used for a rapid reduction of 4-NP and degradation of MB in the presence of NaBH_4_. This successful synthesis and catalytic applications of the green-synthesized AgNPs suggests the potential of using this method as an alternative to the chemical methods in large-scale synthesis, other applications such as electronics, and fabrication of other nanoparticles. The availability, cost-effectiveness compared to other plant sources, and edible properties of *Piper chaba* are the significant advancements of this work.
